# Effect of chlorhexidine pretreatment on clinical performance of adhesive restorations: a systematic review and meta-analysis

**DOI:** 10.3389/fdmed.2026.1746184

**Published:** 2026-03-09

**Authors:** Rim Bourgi, Ahmed A. Holiel, Carlos Enrique Cuevas-Suárez, Miguel Ángel Fernández-Barrera, Evandro Piva, Louis Hardan, Tatiana Roman, Naji Kharouf, Youssef Haikel

**Affiliations:** 1 Department of Biomaterials and Bioengineering, INSERM UMR_S 1121, University of Strasbourg, Strasbourg, France; 2 Department of Restorative and Esthetic Dentistry, Faculty of Dental Medicine, Saint-Joseph University of Beirut, Beirut, Lebanon; 3 Department of Restorative Sciences, Faculty of Dentistry, Beirut Arab University, Beirut, Lebanon; 4 Department of Conservative Dentistry, Faculty of Dentistry, Alexandria University, Alexandria, Egypt; 5 Dental Materials Laboratory, Academic Area of Dentistry, Autonomous University of Hidalgo State, San Agustín Tlaxiaca, Mexico; 6 Department of Restorative Dentistry, School of Dentistry, Federal University of Pelotas, Pelotas, Brazil; 7 Department of Prosthetic Dentistry, Faculty of Dental Medicine, Strasbourg University, Strasbourg, France; 8 Pôle de Médecine et Chirurgie Bucco-Dentaire, Hôpital Civil, Hôpitaux Universitaire de Strasbourg, Strasbourg, France; 9 Department of Endodontics and Conservative Dentistry, Faculty of Dental Medicine, University of Strasbourg, Strasbourg, France

**Keywords:** adhesive restorations, chlorhexidine, clinical performance, dentin bonding, matrix metalloproteinase inhibition, resin–dentin interface

## Abstract

**Statement of problem:**

The long-term stability of resin–dentin adhesion remains a major concern in restorative dentistry. Hydrolytic and enzymatic degradation of the hybrid layer contributes to the deterioration of resin-bonded restorations. Inhibition of matrix metalloproteinases (MMPs) by chlorhexidine digluconate (CHX) has been proposed to preserve the hybrid layer and improve adhesive durability.

**Purpose:**

This systematic review and meta-analysis evaluated the effect of dentin pretreatment with CHX on the clinical performance and longevity of resin-based restorations compared with no CHX application.

**Material and methods:**

Following Preferred Reporting Items for Systematic Reviews and Meta-Analyses (PRISMA) guidelines, electronic searches were conducted in PubMed, Scopus, Web of Science, and the Cochrane Library up to September 2025. Randomized and non-randomized clinical trials assessing CHX pretreatment before adhesive application were included. Primary outcomes were restoration retention, postoperative sensitivity, and secondary caries after ≥6 months of follow-up. Data extraction and risk of bias assessment were performed independently using the Cochrane RoB 2.0 tool. Random-effects meta-analyses were performed using risk differences (RDs) with 95% confidence intervals (CIs).

**Results:**

Eleven clinical trials (6 months–4 years of follow-up) met the inclusion criteria. CHX concentrations ranged from 0.2% to 2% with application times of 15–30 s. Most studies used etch-and-rinse adhesives. Pooled analyses showed no significant differences between CHX and control groups for restoration retention (RD = 0.01; 95% CI, −0.02 to 0.04; *p* = .53), postoperative sensitivity (RD = 0.01; 95% CI, −0.04 to 0.06; *p* = .67), or secondary caries (RD = –0.00; 95% CI, −0.03 to 0.03; *p* = .80), with no heterogeneity (I^2^ = 0%).

**Conclusions:**

Although CHX demonstrates MMPs inhibition *in vitro*, current clinical evidence does not support its routine use for enhancing the longevity of resin–dentin bonds, particularly with etch-and-rinse adhesive systems. CHX may still be useful for antimicrobial purposes or in high-caries-risk patients. Further standardized, long-term randomized clinical trials are warranted to clarify its potential benefits with self-etch and universal adhesives.

**Systematic Review Registration:**

PROSPERO CRD420251165239.

## 1 Introduction

The long-term clinical success of adhesive restorations depends on the stability of the resin–dentin interface. Composite resins provide esthetic and conservative treatment options, but their durability is limited by hydrolytic and enzymatic degradation of the hybrid layer (HL), resulting in loss of retention, marginal discoloration, secondary caries, and postoperative sensitivity (1–3).

In recent years, research has increasingly focused on strategies to enhance the long-term durability of the bond between dental adhesive systems and dentin, as the HL gradually deteriorates due to collagen fiber hydrolysis, even in the absence of bacterial challenge (3, 4).

Matrix metalloproteinases (MMPs) and cysteine cathepsins within dentin are activated during acid etching or by acidic monomers in self-etch adhesives, leading to collagen breakdown within the HL (4, 5). Inhibiting these endogenous enzymes has been proposed as a strategy to enhance long-term bond stability (6).

Chlorhexidine digluconate (CHX) is a well-established cationic bisbiguanide with strong antimicrobial and protease-inhibiting properties (7–10). Due to its substantivity and affinity for dentin phosphate groups, CHX can suppress MMP-mediated collagen degradation and help preserve HL integrity. *In vitro* studies have shown that low CHX concentrations (0.1%–2%) effectively inhibit MMP activity and maintain resin–dentin bond strength for extended aging periods (11, 12).

In 2010, Breschi et al. (5) reported that the application of 0.2% CHX suppressed MMP activity in demineralized dentin aged for two years and extended the durability of adhesive bonds. Similarly, Loguercio et al. (10) found that CHX treatment maintained higher bond strengths after five years of aging, representing the longest follow-up period reported in the literature regarding CHX-mediated MMP inhibition. More recently, Breschi et al. confirmed that CHX retained anti-proteolytic activity within the hybrid layer even after 10 years of accelerated aging, further supporting its role in hybrid layer protection (11).

Nevertheless, the clinical effectiveness of CHX in preserving restoration longevity remains inconclusive. It was reported that CHX pretreatment did not improve the retention and marginal integrity of dental restorations (13).

Such discrepancies may result from variations in CHX concentration, application time, adhesive system, or follow-up duration. Therefore, this systematic review and meta-analysis aimed to synthesize the available clinical evidence regarding the influence of CHX pretreatment on the longevity and performance of resin-based restorations. The null hypothesis was that CHX pretreatment, regardless of concentration or application protocol, does not significantly affect the clinical outcomes of resin–dentin restorations.

## 2 Materials and methods

This systematic review and meta-analysis was conducted according to the Preferred Reporting Items for Systematic Reviews and Meta-Analyses (PRISMA) guidelines (14) and was prospectively registered in the International Prospective Register of Systematic Reviews [PROSPERO; registration number (CRD420251165239)]. Studies were selected based on the population, intervention, comparison, outcome, and study design (PICOS) framework: population (patients receiving adhesive restorations in vital teeth); intervention (dentin pretreatment with CHX applied either as a solution or incorporated into the adhesive protocol); comparison (no CHX pretreatment or conventional adhesive procedure); outcome (clinical performance measures, including restoration retention, marginal adaptation, marginal discoloration, postoperative sensitivity, and secondary caries); and study design [randomized controlled trials (RCTs) or controlled clinical trials]. Only English full-text publications were included, while duplicates and studies without accessible full text were excluded.

Comprehensive electronic searches were conducted in PubMed, Scopus, Web of Science, and the Cochrane Library from database inception to September 2025. Structured search strategies were tailored for each database using combinations of keywords and Medical Subject Headings (MeSH) related to “chlorhexidine,” “dentin,” “adhesion,” “bond strength,” “resin composite,” and “clinical trial” (Table 1). Reference lists of eligible studies and relevant reviews were also screened to identify additional publications. All retrieved records were imported into a reference manager software program (EndNote 20; Clarivate Analytics) for duplicate removal. Two reviewers independently screened titles and abstracts, followed by full-text assessments; disagreements were resolved through discussion or by consulting a third reviewer.

**Table 1:** Search strategy performed at PubMed and adapted to other databases.

Search	Terms
# 1 (Chlorhexidine)	"Chlorhexidine” OR “chlorhexidine digluconate” OR CHX OR digluconate chlorhexidine*
#2 (Adhesive restorations)	"Dentin bonding” OR “resin–dentin bond” OR “bond strength” OR “dental bonding” OR “resin restoration” OR “adhesive restoration” OR “composite restoration”
#3 (Clinical outcomes)	"Clinical performance” OR “restoration retention” OR “restorative survival” OR “marginal adaptation” OR “marginal integrity” OR “secondary caries” OR “postoperative sensitivity"
# 4	#1 AND #2 AND #3

Studies were included if they met the following criteria: (1) randomized controlled trials or controlled clinical trials; (2) human clinical studies involving vital teeth restored with adhesive restorations; (3) use of chlorhexidine as a dentin pretreatment, either as a solution or incorporated into the adhesive protocol; (4) presence of a control group without chlorhexidine pretreatment; (5) reporting at least one clinical outcome of interest, including restoration retention, marginal adaptation, marginal discoloration, postoperative sensitivity, or secondary caries; (6) a minimum follow-up period of one week; and (7) availability of full-text articles published in English. Studies were excluded if they were: (1) *in vitro*, *in situ*, animal, or laboratory-based studies; (2) observational studies, case reports, case series, reviews, or conference abstracts; (3) studies evaluating non-adhesive restorations or non-vital teeth; (4) studies lacking a comparator group; or (5) publications with inaccessible full texts or insufficient clinical outcome data.

Data were independently extracted by two reviewers (MAF-B and EP) for each study, including author, year, country, study design, CHX type and concentration, adhesive system, restoration procedure, control protocol, follow-up period, clinical evaluation criteria, and reported outcomes. Any discrepancies were resolved by consensus. The methodological quality of RCTs was appraised using the Cochrane Risk-of-Bias 2.0 tool, whereas controlled clinical trials were evaluated using the Joanna Briggs Institute (JBI) checklist for quasi-experimental studies (15). Conflicts were resolved through consensus with a third reviewer (TR and NK). The certainty of evidence for each clinical outcome was evaluated using the GRADE approach (16), considering factors such as study risk of bias, consistency of effect, im-precision, indirectness, and publication bias. Certainty ratings were classified as high, moderate, low, or very low, and detailed justifications for downgrading were reported.

When two or more studies reported comparable outcomes, quantitative synthesis was performed using a software program (Review Manager 5.4.1; The Cochrane Collaboration, Copenhagen, Denmark). Effect sizes were expressed as risk ratios (RRs) with 95% confidence intervals (CIs) for dichotomous variables. To account for the time-dependent nature of clinical outcomes and to explore whether the effect of CHX pretreatment varies with observation length, separate meta-analyses were planned according to follow-up duration. Studies were categorized into short-term (<6 months) and long-term (≥6 months) follow-up periods. For studies reporting multiple follow-up assessments, data from the longest available time point within each category were extracted. When a study contributed data to both short- and long-term analyses (e.g., reporting outcomes at 3 and 12 months), the corresponding time points were included separately in each respective pooled analysis. All analyses were conducted using a random-effects model, and heterogeneity was quantified using the I^2^ statistic. A fixed-effect model was used when heterogeneity was low (I^2^ < 50%); otherwise, a random-effects model was applied.

Although the included studies varied in terms of CHX concentration, application time, adhesive system, and cavity class, meta-analytic pooling was considered appropriate for the following reasons: (1) all studies addressed the same PICOS question and shared a common intervention–comparator framework; (2) the outcome measures (retention, postoperative sensitivity, secondary caries) were defined using standardized and comparable clinical criteria; and (3) preliminary assessment of statistical heterogeneity using the I^2^ statistic revealed no significant heterogeneity (I^2^ = 0%) for any of the pooled outcomes, supporting the combinability of effect estimates. These considerations align with current methodological guidance, which permits pooling in the presence of clinical diversity when statistical heterogeneity is low and the body of evidence is coherent.

## 3 Results

**Figure 1:**
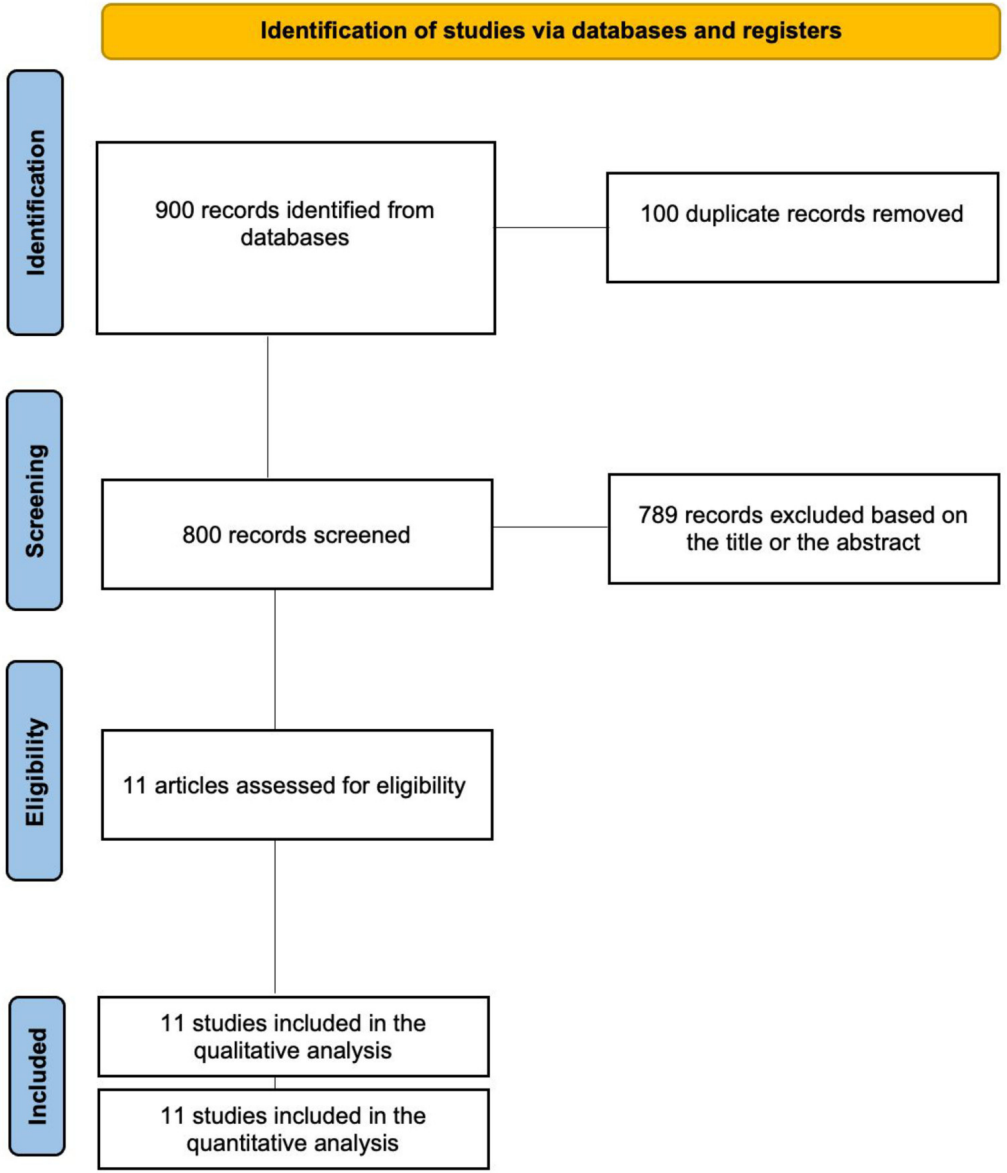
PRISMA flow diagram of study selection.

The electronic search retrieved 900 records from the different databases. After removal of 100 duplicates, 800 titles and abstracts were screened for eligibility. Of these, 789 records were excluded based on the predefined inclusion and exclusion criteria. Following full-text assessment, 11 randomized or controlled clinical trials fulfilled the eligibility criteria and were included in the qualitative synthesis and quantitative analysis (meta-analysis). The study selection process is summarized in the PRISMA flow diagram (Figure 1).

The clinical trials, published between 2005 and 2025, were conducted across diverse populations and settings. Sample sizes ranged from 14 to 60 participants, covering age groups from young children to older adults. Both primary and permanent teeth were included, with cavity classes ranging from non-carious cervical lesions (NCCLs) and Class V restorations to Class I and II carious lesions. CHX was most tested at concentrations of 0.2% and 2%, with application times of 15–30 s in most protocols. Adhesive systems included etch-and-rinse, self-etch, and universal adhesives. Resin composites were the predominant restorative material, placed using either incremental or bulk-fill techniques. Control groups generally followed adhesive protocols without CHX pretreatment. Follow-up durations ranged from 1 week to 4 years. Outcomes were assessed according to standardized clinical criteria, such as the modified United States Public Health Service (USPHS) and Fédération Dentaire Internationale (FDI) evaluation systems, and in some studies supplemented by radiographic examinations, pulp vitality testing, or patient-reported sensitivity assessments. Follow-up durations ranged from 1 week to 4 years, with most studies reporting outcomes at ≥6 months. For meta-analysis, outcomes were grouped into short-term (<6 months) and long-term (≥6 months) follow-up where applicable. The study by Altıntop et al. (25), while reporting up to 12-month data, contributed to the short-term analysis based on its 3-month evaluations. All studies reported at least one of the prespecified clinical outcomes, including restoration retention, postoperative sensitivity, or secondary caries. A summary of the study characteristics is presented in Table 2.

**Table 2:** Characteristics of the studies included in the review.

Author and year	Type of clinical trial	Clinical trial registration	Number (age) of restorations	Type of teeth (temporary/permanent)	Class of cavity	Restoration procedures (Adhesive and resin composite used)	Chlorhexidine application parameters	Evaluation criteria	Follow-up	Key Findings
Sobral et al., 2005 (17)	RCT, split mouth	Not reported	51 restorations, 17 patients (20–48 y)	Permanent premolars	Class II	Control (*n* = 17): 37% phosphoric acid etching + Primer/Bond-1 adhesive applied in two layers for 10 s, air-thinned for 10 s, light-cured 10 s; flowable composite liner (Flow-It) on gingival wall, light-cured 40 s; condensable composite (Alert Condensable) placed in single increments light-cured 40 s per surface. Group 1 – Gluma (*n* = 17): Same as Control + Gluma Desensitizer applied for 1 min before adhesive. Group 2 – Cav-Clean (*n* = 17): Same as Control + Cav-Clean applied for 1 min before adhesive.	Cav-Clean (CHX): applied to cavity walls with cotton roll for 1 min; excess removed with dry cotton pellets	Patient-reported postoperative sensitivity (cold, heat, sweet, floss), 4-point scale	Day 1, Day 4, Day 7	Gluma desensitizer and CHX showed no significant benefit in reducing postoperative sensitivity compared with adhesive alone.
Hajizadeh et al., 2013 (18)	RCT, crossover, double-blind	IRCT201104236267N1	60 restorations, 30 patients (20–35 y)	Permanent premolars	OM Class II	Control (*n* = 30): 35% phosphoric acid etching (enamel 10 s; dentin 5 s) + Single Bond two-step adhesive; universal microhybrid composite (Filtek Z250) applied incrementally Experimental (*n* = 30): Same as Control + 2% CHX aqueous solution (Concepsis, Ultradent), 60 s before adhesive	2% CHX solution applied to etched dentin for 60 s; air-dried; applied as separate cavity disinfectant	Patient-reported postoperative sensitivity (VAS 11-point), categorized	Baseline, Day 1, 1 week, 1 month, 6 months*	CHX significantly reduced immediate postoperative sensitivity at 1 day, with no differences at later follow-ups.
Dutra-Correa et al., 2013 (12)	RCT	Not reported	120 restorations, 37 patients (27–79 y)	Permanent premolars, molars	Class V	Group 1 – XP Bond (XPB, *n* = 30): Two-step etch-and-rinse adhesive; restored with Esthet•X composite in 1–1.5 mm increments; each increment light-cured for 20 s Group 2 – CHX + XPB (*n* = 30): Same + 2% CHX post-etch. Group 3 – Xeno V (XEN, *n* = 30): One-step self-etch adhesive; Esthet•X composite as above. Group 4 – CHX + XEN (*n* = 30): Same + 2% CHX pre-adhesive	2% CHX aqueous solution applied to dentin surface after etching (for XPB) or prior to self-etch adhesive (XEN) for 60 s; air-dried; applied separately	Modified UNC-USPHS: retention, color match, marginal staining, wear, adaptation, texture, pre-/post-op sensitivity	Baseline, 1 week, 6 month*, 18 month^§^	CHX pretreatment did not affect 6- or 18-month clinical performance or retention of either adhesive system.
Sartori et al., 2013 (19)	RCT, crossover	Not reported	70 restorations, 20 patients (33–64 y)	Permanent incisors, canines, premolars, molars	Class V	Control Group – Adper Single Bond 2 (SB, *n* = 35): Two-step etch-and-rinse adhesive; restored with Filtek Supreme XT nanofilled composite in three increments, gingival margin first; each increment cured for 20 s. Experimental Group – CHX + SB (*n* = 35): Same as control, with prior application of 2% CHX digluconate after acid etching and before adhesive.	2% CHX digluconate applied to dentin after etching under scrubbing action for 30 s; excess removed with cotton pellets	Modified USPHS: retention, marginal discoloration/integrity, post-op sensitivity, caries, pulp vitality	Baseline, 6 months*, 12 months, and 36 months^§^	CHX pretreatment did not improve long-term clinical performance or durability of Class V adhesive restorations.
Araújo MS. Et al., 2015 (20)	Double-blind, RCT	Local Ethics Committee (protocol 95/10)	88 restorations, 22 patients (45 ± 8 y)	Permanent anterior and posterior teeth	Class V	Control groups: CSE (Clearfil SE Bond) or ADS (AdheSE) applied per manufacturer instructions; composite: Filtek Z-250 in 2–3 increments of 2 mm, light-cured 20 s each, Experimental groups: CSE/CHX or ADS/CHX, then same adhesive and restoration procedure as control.	50 μL of 20% CHX digluconate added to 950 μL primer to yield 1.0 wt% CHX; applied as primer before adhesive	Modified USPHS: retention, marginal staining/adaptation, post-op sensitivity, caries recurrence	Baseline and 24 months^§^	Incorporation of CHX did not improve 2-year clinical performance; Clearfil SE Bond showed higher retention than AdheSE regardless of chlorhexidine.
Montagner et al., 2015 (13)	Prospective RCT, split-mouth, triple-blind	NCT01947192	169 restorations, 42 patients (21–76 y)	Permanent incisors, canines, premolars	Class V	Group1: 35% phosphoric acid etching (dentin 20 s, enamel 15 s) + Adper Single Bond 2 adhesive, followed by Filtek Z350 nanocomposite resin in ≥2 increments (≤2 mm thick), each light-cured 20 s at 800 mW/cm^2^. Group2: Same + 2% CHX, 60 s post-etch Control: Same + Placebo solution applied for 60 s post-etch	2% CHX applied 60 s post-etch, excess removed	FDI: retention, marginal adaptation/staining, surface staining, post-op sensitivity, aesthetics, fracture, anatomy, vitality	6 months*	CHX application did not affect 6-month retention of Class V restorations; cavity geometry, not CHX, influenced failure.
Galafassi et al., 2017 (21)	Prospective RCT, split mouth	FORP/USP—Case No 02937212.5.0000.5419	80 restorations, 20 children (8–12 y)	Permanent molars	Class I (occlusal)	Group1: 37% phosphoric acid (enamel 30 s, dentin 15 s) + Adper Single Bond 2, light-cured 10 s. Restoration: Filtek Z350 XT incremental composite). Group 2: same + 2% CHX, 60 s post-etch Control: deionized water applied to dentin for 60 post-etch	2% CHX applied to dentin for 60 s after acid etching; excess removed. s.	Modified USPHS criteria (retention, marginal adaptation, marginal discoloration, other failures);	Baseline, 6 months*, 12 months^§^	Laser-prepared cavities showed superior clinical performance regardless of re-wetting agent; CHX did not significantly influence outcomes.
Favetti et al., 2017 (22)	RCT, crossover	NCT01947192	105 restorations, 42 patients (49.7 y)	Permanent incisors, canines, premolars	Class V	Group1: Two-step etch-and-rinse adhesive (Single Bond 2, 3M ESPE) applied after 35% phosphoric acid etching (enamel 20 s, dentin 15 s), light-cured 10 s; restored with Filtek Z350 nanocomposite resin in 2 increments ≤2 mm thick, light-cured 20 s per increment. Group 2: CHX + SB: same + 2% CHX, 60 s Control: placebo solution applied in the same manner	2% CHX applied to etched dentin for 60 s with scrubbing, excess removed	FDI: retention, marginal adaptation/staining, post-op sensitivity, surface properties, fracture, anatomy, vitality	Baseline (1 wk), 6 months*, 12 months, 24 months, and 36 months^§^	CHX pretreatment did not improve long-term retention or survival of NCCL restorations over 36 months.
Valério RA et al. 2020 (23)	RCT, split-mouth	ReBEC RBR-8n5n3v	64 restorations, 16 patients (8–12 y)	Permanent first molars	Class I	Control (*n* = 16): Selective caries removal with bur or Er:YAG laser; 100% nanoparticle composite containing zirconia and silica; adhesive applied; restorations placed in increments ≤2 mm and light-cured Group 1 – same + 2% CHX applied post-caries removal Group 2 same + deionized water using same protocol	2% CHX applied after caries removal, before adhesive; excess removed	Modified USPHS: retention, marginal adaptation/discoloration, secondary caries, pulp vitality	Baseline, 6 months*, 12 months, 48 months^§^	Er:YAG laser or bur preparation produced similar 4-year restoration survival; CHX biomodification did not influence outcomes.
Nemt-Allah AA 2022 (24)	RCT	NCT03669224	57 restorations, 19 patients (17–50 y)	Permanent posterior teeth	Class II	Control (*n* = 19): Scotchbond Universal Etchant on enamel 15 s, rinsed 15 s, dentin protected with wet cotton; Single Bond Universal adhesive applied 20 s, air-thinned 5 s, light-cured 10 s; Filtek Z350 XT packable composite inserted using incremental oblique layers, 20 s per increment Group 1 – NanoCare Gold (*n* = 19): same + 5 coats NanoCare Gold, 3 min each, solvent allowed to evaporate naturally. Group 2 – same + 2% CHX, 1 min	2% CHX: Consepsis applied 1 min, excess removed; separate step	FDI criteria for marginal adaptation, marginal staining, and postoperative sensitivity	Immediate, 6 months*, 12 months*, and 24 months^§^	No significant differences were observed among groups; CHX did not affect marginal adaptation, staining, or postoperative sensitivity for up to 2 years.
Altıntop H., et al. 2025 (25)	Prospective, single-blind, RCT	NCT06257108	80 restorations, 40 patients (5–9 y)	Primary molars	Class II	Control (*n* = 40): 37% phosphoric acid etching + PQ1 Bond applied for 10 s, air-dried 10 s, light-cured 10 s; Tetric N-Ceram Bulk Fill Composite applied in horizontal layers up to 4 mm, light-cured 10 s per layer Experimental group (*n* = 40): Same + Peak Universal Bond with 0.2% CHX	CHX incorporated within Peak Universal Bond (0.2%), no separate application step	FDI: esthetic, functional, biological properties, post-op sensitivity, secondary caries, retention, marginal adaptation	3 months, 6 months*, 9 months, and 12 months^§^	CHX-containing adhesive showed similar 12-month clinical success to standard adhesive and higher microtensile bond strength, without compromising performance.

Data used for the meta-analysis subgroup for comparison up to 6 months of follow-up is indicated with the symbol *. Where multiple follow-up assessments were available, the time point used in the primary meta-analysis (≥ 6 months follow-up) is indicated with the symbol §.

**Figure 2:**
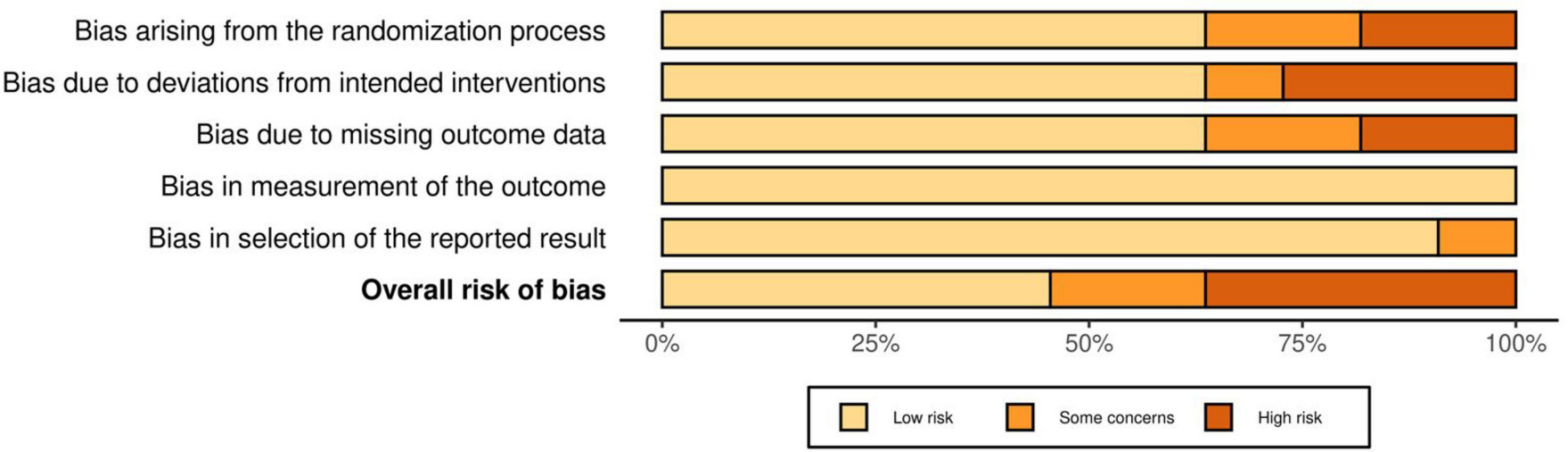
Risk of bias graph: review of authors’ judgments about each risk of bias item presented as percentages across all included studies. Domains: D1: Bias arising from the randomization process; D2: Bias due to deviations from intended interventions; D3: Bias due to missing outcome data; D4: Bias in measurement of the outcome; D5: Bias in selection of the reported result.

**Figure 3:**
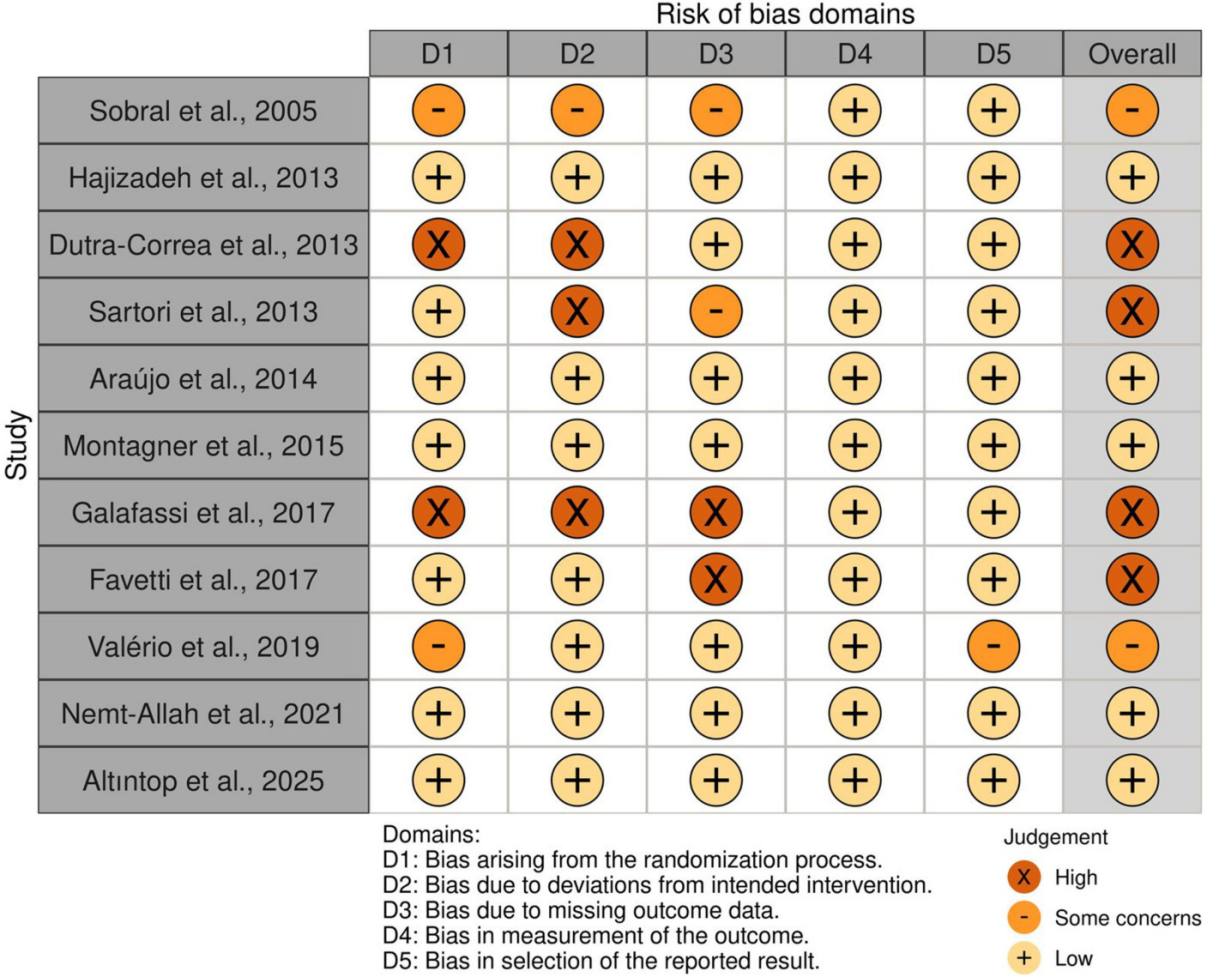
Risk of bias summary: review of authors’ judgments about each risk of bias item for each included study. Symbols: “+” Low risk; “-” Some concerns; “X” High risk.”.

The risk of bias assessment indicated that most studies presented low to moderate risk across the five domains of the Cochrane RoB 2.0 tool. Some studies had unclear allocation concealment, incomplete outcome data, or potential deviations from intended interventions; however, overall confidence in the results was considered acceptable (Figures 2, 3).

The certainty of evidence for the clinical outcomes associated with dentin pretreatment with chlorhexidine (CHX) was evaluated using the GRADE approach. Evidence derived from 11 randomized and controlled clinical trials provides moderate-certainty evidence that CHX pretreatment does not improve the clinical performance of adhesive restorations compared with conventional adhesive protocols without CHX, with respect to restoration retention, postoperative sensitivity, and secondary caries incidence (Table 3). Downgrading of the evidence was primarily driven by imprecision, related to modest sample sizes and the limited number of clinical events, as well as some concerns regarding risk of bias in individual studies, including incomplete reporting of allocation concealment, blinding, or outcome data. Across outcomes, the direction and magnitude of effects were consistent, and statistical heterogeneity was negligible, indicating no serious concerns regarding inconsistency. Indirectness was not considered serious, as the populations, interventions, comparators, and outcomes were directly relevant to contemporary clinical adhesive dentistry. For secondary caries incidence, the certainty of evidence was rated as low, owing to very few reported events and relatively short follow-up periods, resulting in substantial uncertainty around the effect estimates. Overall, the available evidence indicates that CHX dentin pretreatment results in short- to medium-term clinical performance comparable to standard adhesive procedures, without conferring a measurable advantage in restoration longevity or biological outcomes.

**Table 3:** GRADE summary of evidence for randomized controlled trials evaluating chlorhexidine dentin pretreatment.

Clinical outcome	No. of studies (restorations)	Study design	Risk of bias	Inconsistency	Indirectness	Imprecision	Publication bias	Overall certainty of evidence (GRADE)
Restoration retention (≥6 months)	9 (∼833)	RCTs	Not serious	Not serious	Not serious	Serious^a^	Not serious	⊕⊕⊕⊝ Moderate
Postoperative sensitivity	10 (∼880)	RCTs	Not serious	Not serious	Not serious	Serious^a^	Not serious	⊕⊕⊕⊝ Moderate
Secondary caries incidence	4 (∼302)	RCTs	Not serious	Not serious	Not serious	Very serious^b^	Not serious	⊕⊕⊝⊝ Low

^a^ Serious imprecision: Several trials had modest sample sizes and low numbers of clinical events; confidence intervals were compatible with both no effect and small clinically relevant benefit or harm.

^b^ Very serious imprecision: Secondary caries events were infrequent, and follow-up durations limited, resulting in wide plausible effect ranges and substantial uncertainty.

**Figure 4:**
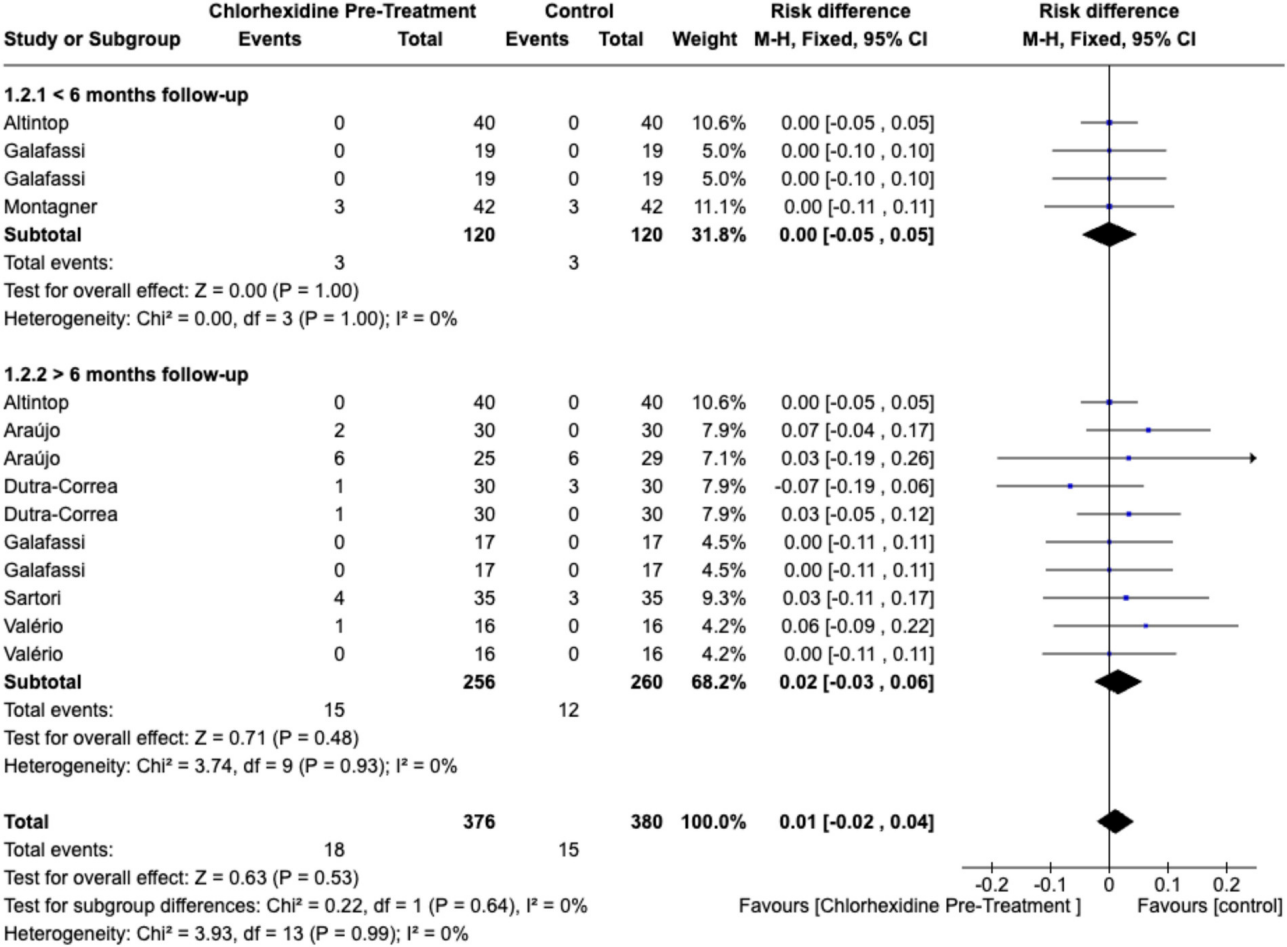
Forest plot of restoration retention comparing CHX pretreatment vs. control groups. Data for ≥6 months follow-up represent the longest available follow-up for each study. Comparator groups are placebo (water) controls where available; otherwise, standard adhesive-only controls were used.

For restoration retention, pooled analysis showed no statistically significant difference between CHX-treated and control groups at either short-term (< 6 months) or long-term (>6 months) follow-up (*p* > .05). The combined estimate indicated comparable retention rates (risk difference = 0.01; 95% CI, −0.02 to 0.04; *p* = .53) with no heterogeneity (I^2^ = 0%) (Figure 4).

**Figure 5:**
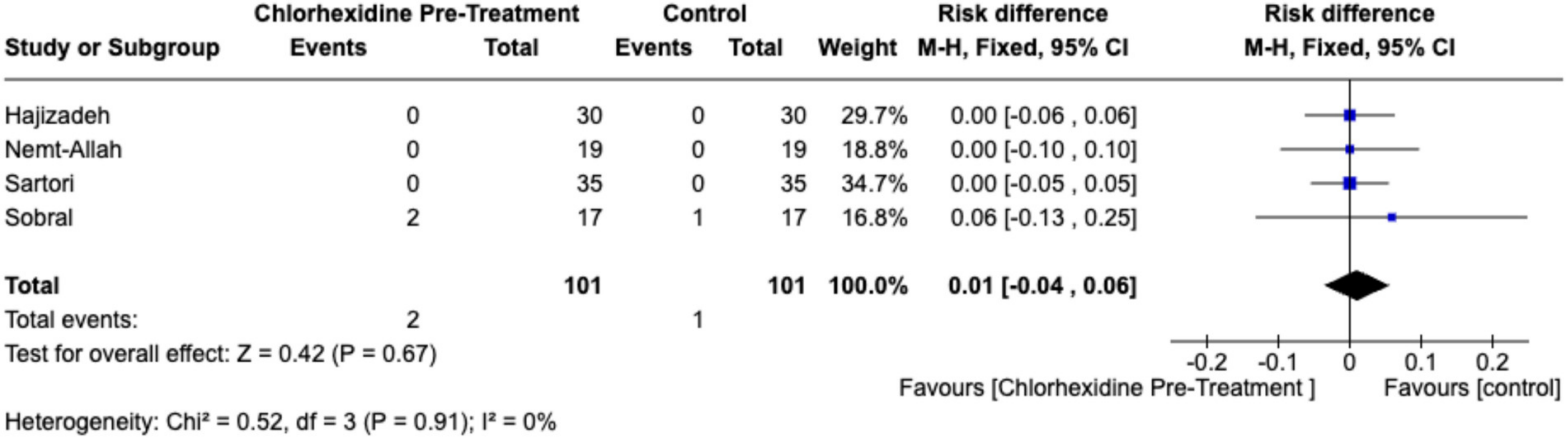
Forest plot of postoperative sensitivity comparing CHX pretreatment vs. control groups. Data for ≥6 months follow-up represent the longest available follow-up for each study. Comparator groups are placebo (water) controls where available; otherwise, standard adhesive-only controls were used.

**Figure 6:**
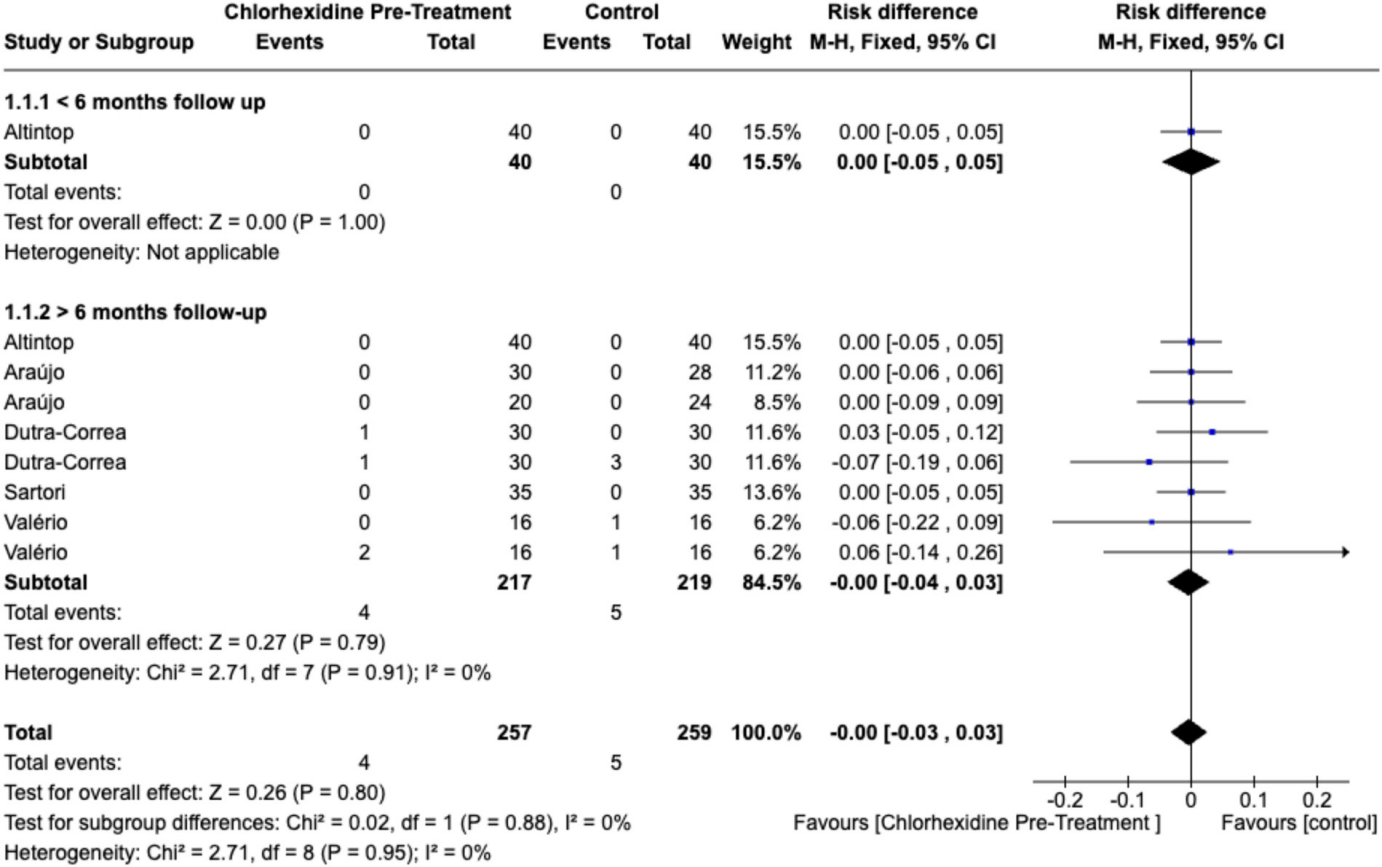
Forest plot of secondary caries comparing CHX pretreatment vs. control groups. Data for ≥6 months follow-up represent the longest available follow-up for each study. Comparator groups are placebo (water) controls where available; otherwise, standard adhesive-only controls were used.

Similarly, no significant difference was found in postoperative sensitivity (RD = 0.01; 95% CI, −0.04 to 0.06; *p* = .67; I^2^ = 0%) (Figure 5) or in secondary caries incidence (RD = 0.00; 95% CI, −0.03 to 0.03; *p* = .80; I^2^ = 0%) (Figure 6).

## 4 Discussion

This systematic review and meta-analysis critically examined the clinical effects of CHX pretreatment on adhesive restorations, focusing on restoration retention, postoperative sensitivity, and secondary caries. Across 11 randomized clinical trials, CHX application, whether used as a cavity disinfectant or after acid etching, did not significantly affect clinical performance compared with conventional adhesive protocols. These findings support the null hypothesis and indicate that CHX pretreatment offers no additional long-term clinical advantage in routine adhesive dentistry.

Mechanistically, CHX exhibits high affinity for demineralized dentin due to its cationic nature and capacity to bind phosphate groups, resulting in durable substantivity and inhibition of MMPs responsible for hybrid layer degradation (26). Although laboratory studies demonstrate sustained bond strength preservation through MMP inhibition (27–29), the clinical evidence remains inconsistent. This discrepancy may be partly explained by the fact that the beneficial effect of CHX is markedly diminished when applied to mineralized dentin prior to etching, due to its limited penetration and binding capacity. This may explain the poor translation of *in vitro* benefits into clinical performance, as highlighted by Frassetto et al. (30).

Clinically, most included trials used etch-and-rinse adhesives (e.g., AdheSE, XP Bond, Single Bond Universal, Bond, Peak Universal Bond), which establish durable hybrid layers and chemically interact with hydroxyapatite through functional monomers such as 10-methacryloxydecyl dihydrogen phosphate (10-MDP) (31, 32). The inherent chemical stability and micromechanical retention of these systems likely overshadow any incremental benefit from CHX-mediated MMP inhibition, consistent with the stable retention outcomes observed across follow-ups of up to four years (22, 24).

It should be noted that one included study evaluated primary teeth (25), while the others focused on permanent dentition. Although the dentin structure and mineralization differ, the consistent findings (with no statistical heterogeneity) across both populations in our analysis suggest that, within the observed follow-up periods, the clinical effect of CHX pretreatment may not be significantly influenced by dentition type. However, this observation is based on a single pediatric study and warrants confirmation in future research.

The reviewed studies varied in CHX concentration and application protocols, typically using 0.2% to 2% for 15–60 s after etching (13, 18, 20, 25). Shorter application times or lower concentrations may limit CHX diffusion into demineralized dentin, reducing its inhibitory potential. Additionally, universal adhesives contain hydrophilic solvents and acidic monomers that could partially remove or dilute CHX residues, altering its interaction with collagen fibrils (25). Despite these variations, meta-analytic results revealed no significant heterogeneity (I^2^ = 0%), reinforcing the robustness and reproducibility of the overall clinical trend.

Several clinical and material-related factors may further explain the limited effect of CHX *in vivo*. Continuous mechanical stresses from occlusal loading, polymerization shrinkage, and thermal and pH fluctuations induce fatigue and degradation of the resin–dentin interface, diminishing the influence of enzymatic inhibition over time (31). Differences in cavity configuration, restoration type, oral environment, and patient-specific factors such as hygiene and dietary habits also contribute to interstudy variability. These complex clinical dynamics, absent in controlled laboratory conditions, likely explain the discrepancy between the biochemical efficacy of CHX and its limited clinical impact.

It is worth noting that other enzymatic inhibitors, such as benzalkonium chloride (BAC), quaternary ammonium methacrylates (QAMs), or natural extracts (e.g., proanthocyanidins), have also been investigated for their potential to stabilize the resin–dentin interface (33–35). These agents may offer different mechanisms of action or substantivity profiles compared to CHX. Future clinical research could benefit from direct comparisons between CHX and these alternative inhibitors to identify the most effective strategy for enhancing bond durability in specific clinical scenarios.

The methodological quality of the included studies was generally acceptable, with most trials demonstrating low to moderate risk of bias. While allocation concealment and examiner blinding were adequately reported, some studies lacked detailed descriptions of randomization or dropout management. Variations in restoration class, adhesive type, and evaluation criteria could also introduce subtle clinical heterogeneity not fully captured by statistical models. Standardized protocols and long-term randomized clinical trials are needed to determine whether CHX pretreatment may offer measurable benefits with self-etch or universal adhesives, which are more prone to hydrolytic and enzymatic degradation. In addition, Embase and gray literature were not systematically searched due to access limitations and the review's focus on peer-reviewed clinical trials; however, manual reference screening was performed to minimize the risk of missing relevant studies. Despite these limitations, CHX application remains a safe adjunctive procedure that does not compromise bonding effectiveness. The consistency of clinical outcomes across adhesive systems and CHX concentrations indicates that current adhesives provide reliable long-term performance, even in the absence of MMP inhibition strategies. Therefore, based on current evidence, CHX pretreatment should not be considered a mandatory step in routine adhesive protocols. However, clinicians may still consider it for its antimicrobial and disinfectant properties, especially in patients with high caries risk or in cases requiring additional dentin decontamination for selective preventive purposes.

## 5 Conclusions

Based on the available clinical evidence dentin pretreatment with CHX did not show a significant effect on the clinical performance of resin-based restorations in terms of retention, postoperative sensitivity, or secondary caries. While CHX is an effective MMP inhibitor *in vitro*, this benefit was not translated into a measurable clinical advantage in the reviewed trials. Broad conclusions regarding CHX's clinical inefficacy should be avoided due to the constrained evidence base. Further long-term, standardized RCTs are warranted to clarify its potential role, particularly with modern adhesive systems.

## Data Availability

The raw data supporting the conclusions of this article will be made available by the authors, without undue reservation.
